# EHMTI-0139. Field-testing of the ICHD-3 beta diagnostic criteria for classical trigeminal neuralgia

**DOI:** 10.1186/1129-2377-15-S1-C38

**Published:** 2014-09-18

**Authors:** S Maarbjerg, MT Sørensen, A Gozalov, L Bendtsen, J Olesen

**Affiliations:** 1 Danish Headache Center Department of Neurology, Glostrup Hospital University of Copenhagen, Glostrup, Denmark; 2 Faculty of Health and Medical Sciences, University of Copenhagen, Glostrup, Denmark

## Introduction

In the summer of 2013 the International Headache Society (IHS) published the beta-version of the 3rd International Classification of Headache Disorders (ICHD-3 beta) with revised diagnostic criteria for classical trigeminal neuralgia (TN). The TN diagnostic criteria are based on expert opinion and IHS strongly encourages field-testing of the new diagnostic criteria.

## Aims

We aimed to field-test ICHD-3 beta diagnostic criteria for TN by comparing sensitivity and specificity to ICHD-2 criteria, and evaluate needs for revision.

## Methods

Clinical characteristics were systematically and prospectively collected from 206 consecutive TN patients and from 37 consecutive patients with persistent idiopathic facial pain in a cross-sectional study design. We used a modified version of the 2nd International Classification of Headache Disorders (ICHD-2) to allow for sensory abnormalities. Symptomatic trigeminal neuralgia and posttraumatic painful trigeminal neuropathy were excluded based on a thorough history and 3.0 Tesla MRI.

## Results

The specificity of ICHD-3 beta was similar to ICHD-2 (97.3% vs. 89.2%, p = 0.248) and the sensitivity was unchanged (76.2% vs. 74.3%, p = 0.134). The majority of false negative diagnoses in TN patients were due to sensory abnormalities. With a proposed modified version of ICHD-3 beta it was possible to increase sensitivity to 96.1% (p < 0.001 compared to ICHD-3 beta) while maintaining a specificity at 83.8% (p = 0.074 compared to ICHD-3 beta).

## Conclusions

ICHD-3 beta was not significantly different from ICHD-2 and both lacked sensitivity. A modification of the criteria improved the sensitivity greatly and is proposed for inclusion in the forthcoming ICHD-3.

No conflict of interest.

